# Editorial: Insights in structural interventional cardiology: 2022

**DOI:** 10.3389/fcvm.2023.1305452

**Published:** 2023-11-01

**Authors:** Diego Arroyo, Peter Martin Wenaweser

**Affiliations:** ^1^ Department of Cardiology, University and Hospital Fribourg, Fribourg, Switzerland; ^2^ Department of Cardiology, Heart Clinic Zurich, Zurich, Switzerland; ^3^ Department of Cardiology, Swiss Cardiovascular Center Bern, University Hospital Bern, Bern, Switzerland

**Keywords:** TAVI (transcatheter aortic valve implantation), stroke, STEMI, radial access, valve in valve transcatheter aortic valve implantation, bicuspid aortic stenosis

**Editorial on the Research Topic** Insights in structural interventional cardiology: 2022

Since the inception of percutaneous coronary balloon angioplasty in 1977, interventional cardiology has predominantly revolved around coronary interventions. However, the landscape of interventional cardiology has evolved significantly with the introduction of coronary stenting and select structural interventions like PFO closure and mitral valvuloplasty. The treatment of valvular heart conditions, historically dominated by open-heart surgical procedures, witnessed a transformative shift with the advent of transcatheter aortic valve replacement (TAVI) in 2002.

Catheter-based technologies have since emerged as a vital therapeutic option, particularly for elderly, high-risk patients. Today, modern valve replacements and repair approaches primarily rely on catheter-based techniques, promising minimally invasive procedures that appeal to both patients and dedicated medical practitioners. This evolution has given rise to a dynamic community of surgeons and cardiac interventionalists collaborating closely with industry leaders, fostering innovation and investments in this burgeoning field. The field of cardiovascular medicine has made remarkable strides in recent years, and as we reflect upon the year's achievements, it becomes clear that our understanding and approach to cardiovascular care are continually evolving.

In this editorial, we summarize the most viewed and downloaded articles of the year 2022 within the Research Topic “Structural Interventional Cardiology” from the journal Frontiers in Cardiovascular Medicine. As we explore these influential studies, we find ourselves at the intersection of groundbreaking research and innovative approaches, shedding light on crucial topics that shape the field. These studies represent the dedication and innovation of researchers worldwide to improve patient outcomes and enhance our understanding of cardiovascular diseases.

Transcatheter aortic valve implantation (TAVI) has revolutionized the treatment of aortic valve disease. However, it comes with a challenge—periprocedural strokes. The review provided by Linder and Seiffert delves into this critical issue (Linder and Seiffert). Stroke during or after TAVI remains a serious complication, with an incidence of 2%–3% at 30 days. As TAVI extends to younger, low-risk patients, the prevention of periprocedural strokes becomes even more crucial. While various cerebral embolic protection devices have been tested, a clear clinical benefit has not been demonstrated in randomized trials. This study emphasizes that stroke prevention should encompass different procedural and periprocedural strategies, recognizing the multifactorial nature of stroke etiology. The optimal antithrombotic, anticoagulation therapy with or without the use of a cerebral protection device remain to be evaluated in future clinical trials.

Another challenge in TAVI procedures is the correct positioning of the valve with respect to access to the coronary ostia. Especially in patients with bicuspid aortic stenosis the anatomy varies widely. In the work of Tarantini et al., a novel technique based on the coronary ostia overlap rather than the cusp overlap view has been evaluated and controlled with CT scan (Tarantini et al.). It appears that this novel, sophisticated approach might be more accurate and safe regarding the goal of providing coronary access, especially after implantation of a self-expanding prosthesis.

The Radial Artery Puncture Hemostasis Evaluation (RAPHE) study, led by Kulyassa et al., will explore a novel approach—non-compression-based radial artery hemostasis methods (Kulyassa et al.). This prospective, multicenter clinical trial investigates the efficacy and safety of two such methods, a chitosan bioactive hemostatic dressing and a purpose-built radial potassium-ferrate based topical hemostasis disc. By challenging the conventional compression-based approach, this study offers a promising avenue to enhance patient care and procedural safety.

Transcatheter aortic valve implantation (TAVI) has become a standard of care for patients with aortic valve disease. However, there are concerns about new adverse coronary events (NACE) especially in valve-in-valve procedures. Koren et al.'s research investigates the incidence of adverse events following valve-in-valve (Koren et al.). The study uses data from a substantial cohort of TAVI patients and propensity-matching to compare outcomes. The results show a higher in-hospital coronary artery obstruction rate with valve-in-valve procedures, aligning with previous reports. However, despite this, the 2-year cumulative NACE rates were similar between groups, including myocardial infarction, unplanned coronary catheterization, or coronary artery bypass grafting. Importantly, the study identifies factors that influence outcomes, such as coronary artery height, providing clinicians with valuable information for patient selection and management.

Enhancing patient care while optimizing healthcare efficiency is a constant goal in the field of cardiovascular medicine. The study by Koren et al. explores the safety of early discharge following transfemoral TAVI under general anesthesia (Koren et al.). This practice has gained traction, particularly for low-risk patients. By analyzing a large dataset of patients, the study categorizes patients based on the time of discharge, validates predictors for adverse outcomes, and highlights the importance of balancing early discharge with the predicted risk factors at baseline.

In the realm of ST-segment elevation myocardial infarction (STEMI), optimizing treatment strategies is critical, especially for patients at high risk of ischemic events. The study by Lee et al. evaluates the safety and efficacy of contemporary drug-eluting stents (DES) in high-risk STEMI patients (Lee et al.). This research addresses an important clinical question, as the safety and efficacy of DES in this specific patient population have been unclear. By analyzing a large cohort of patients who underwent primary percutaneous coronary intervention (PCI) with everolimus-eluting stents (EES) and zotarolimus-eluting stents (ZES), the study provides insights into tailoring treatment strategies for high-risk STEMI patients, ultimately improving outcomes.

Research plays a pivotal role in advancing our understanding of cardiovascular diseases and refining their treatment approaches. Beyond the articles featured in this review, numerous other high-quality contributions within this topic warrant recognition. These include insightful case reports, comprehensive reviews covering various facets of transcatheter aortic valve implantation (TAVI) and percutaneous mitral procedures, and numerous original articles, collectively enriching the knowledge landscape in structural interventional cardiology. As we look ahead, it is clear, that interventional approaches hold the potential to solve a myriad of structural heart valve problems, thereby further reducing cardiac mortality rates. Our commitment to this remarkable field is unwavering, as we continue to critically review and publish articles that contribute to its growth.

